# Investigating the effects of tilting the postural drainage lithotripsy system on cerebral blood flow, intracranial pressure, heart rate, and blood pressure

**DOI:** 10.1007/s00345-024-04777-w

**Published:** 2024-02-20

**Authors:** Liangliang Dai, Shihui Li, Tao Yang, Hanping Wei, Rijin Song, Xianghu Meng, Xiaoliang Yuan, Zhimin Jiao, Tingchun Wu, Honglei Shi

**Affiliations:** 1 Department of Urology, Wujin Hospital Affiliated With Jiangsu University, Changzhou, 213004 China; 2 Department of Urology, The Wujin Clinical College of Xuzhou Medical University, Changzhou, 213004 China; 3 Wujin Hospital Affiliated With Jiangsu University, Changzhou Wujin People’s Hospital, Changzhou Medical Center, Nanjing Medical University, Changzhou, 213004 China; 4 Department of Urology, The First Affiliated Hospital of Nanjing Medical University, Nanjing, 210029 China

**Keywords:** Postural lithecbole, Minimally invasive, Cerebral blood flow, Intracranial pressure, Transcranial Doppler ultrasound

## Abstract

**Purpose:**

To investigate the effect of the postural drainage lithotripsy system developed by our experimental team on the vital signs of patient with urinary stones during the stone removal process.

**Methods:**

Four groups of 15 subjects (0°, 10°, 40°, and 70°) were subjected to different angles of head-down tilt to measure middle cerebral artery blood flow velocity (MCAv), cerebrovascular conductance coefficient (CVCi), intracranial pressure (nICP), heart rate (HR), and mean arterial blood pressure (MAP).

**Results:**

As the angle of HDT changed, MCAv values, nICP values, CVCi values, HR values, and MAP values changed significantly (all *P* ≤ 0.001), and the difference was statistically significant. During 10°HDT, despite a slight increase in nICP, the other measurements remained stable. During 40°HDT, only the MCAv values did not change significantly, whereas the rest of the measures were significantly altered. During 70°HDT, all indicators changed significantly**.**

**Conclusions:**

The significant alterations in cerebral blood flow, intracranial pressure, and hemodynamics induced during the treatment of renal residual fragments with postural drainage should be used with caution in individuals with cerebrovascular accidents.

**China Clinical Trials Registry:**

ChiCTR2300070671; Registration date: 2023-04-18.

## Introductions

Urolithiasis affects 5 to 15 percent of the world’s population [1, 2]. Despite tremendous advances in the treatment of urinary stones, postoperative residual fragments (RFs) may recolonize and grow, leading to renal colic, infection, and stone recurrence [3]. With patients following minimally invasive urological stone surgery being encouraged to participate in active lithecbole exercise programs, lithecbole using the inverted position has been widely used as a therapeutic modality for RFs [4–7]. However, the efficacy is not satisfactory [8]. The main reason is that the angle of inversion varies from 12°to 45°, and even greater than 60°to 70°, there is no single reliable value and a single plane of inversion angle [9–11], a single plane of inversion cannot change the position of renal anatomy in three-dimensional space, and when encountered with residual stones in the kidney in the angle of the difficult position of the lithecbole effect is greatly reduced. In addition, since the renal collecting system alignment is inconsistent in each individual, the method of simply inverting the patient’s position for stone removal is slightly blind. Therefore, it is necessary to investigate a new physical device and method to explore a more effective, reasonable, and individualized inversion angle to achieve targeted stone removal.

Our research team developed the postural drainage lithotripsy system (PLDS), which individualizes lithecbole by calculating the drainage path of RFs through software and using a lithecbole bed to naturally drain the stone under gravity, which has been validated by in vitro model trials [12]. For real patients, we believe that the lithecbole bed in PDLS does not affect their vital sign status when turned from side to side in Y-axis rotation; however, patient safety in X-axis inversion is debatable.

Some medical procedures place the patient in an inverted or Trendelenburg position, such as the inverted table used for back pain [13] and the Trendelenburg position often used for laparoscopic surgery [14]. However, neurological complications such as cerebral edema [15] or hemiparesis [16] may occur after robotic-assisted laparoscopic surgery for 30° and 45° HDT. The cause of such surgical complications may be elevated intracranial pressure and/or alterations in cerebral circulation. Therefore, various studies focusing on cerebral circulation and intracranial pressure have been carried out in recent years in order to gain a better understanding of these important health issues in the field of space medicine [17, 18]. Related studies have explored the effects on the eye and cardiovascular system during inversion [19, 20], but the results have been contradictory. In one study, there was a correlation between increases in blood pressure, heart rate, and intraocular pressure during inversion [20], while other studies found no clinically significant changes in cardiovascular hemodynamics in healthy populations [19, 21].

This study aimed to evaluate the effects of PDLS on intracranial pressure (ICP), cerebral blood flow (CBF), and hemodynamics of subjects during the treatment of RFs using ultrasound and to confirm its safety. This will help patients with urinary stones to actively remove stones after minimally invasive surgery, further improve the stone free rate (SFR) of RFs after minimally invasive surgery, and reduce the economic burden as well as improve the quality of life of patients.

## Methods

### Study design

Criteria for excluded populations: spinal cord injury, brain injury, glaucoma, respiratory disease, high resting blood pressure (systolic > 160 mmHg, diastolic > 90 mmHg), electrocardiographic abnormalities (S-T suppression, > 3 consecutive ectopic beats), and failure to obtain middle cerebral artery mean flow velocity (MCAv) by transcranial doppler ultrasound (TCD).

A total of 100 subjects were recruited and divided into three groups according to age: < 40 years old, 40–60 years old, and > 60 years old. There were 24 patients in the < 40-year-old group, 37 in the 40–60-year-old group, and 39 in the > 60-year-old group. The patients in each group were numbered sequentially and then a simple random sampling method using a computer was used to select the sample units and for each age group, five patients were randomly selected for the experiment.

Ethical approval for this experiment was obtained from the Wujin Hospital of Jiangsu University (Approval No. 2023-SR-086), and all procedures adhered to the principles of the Declaration of Helsinki. All participants provided written informed consent and medical history regarding cardiovascular health and were screened based on a physical examination including electrocardiogram (ECG) and blood pressure (BP) measurements.

The experiment was conducted over four days, with each participant taking four measurements and measuring the head-down tilt (HDT) angle only once per day. Subjects were instructed to avoid strenuous exercise, smoking, and alcohol consumption for 24 h, to avoid overeating for 4 h, and to rest for 30 min before treatment.

**Fig. 1:**
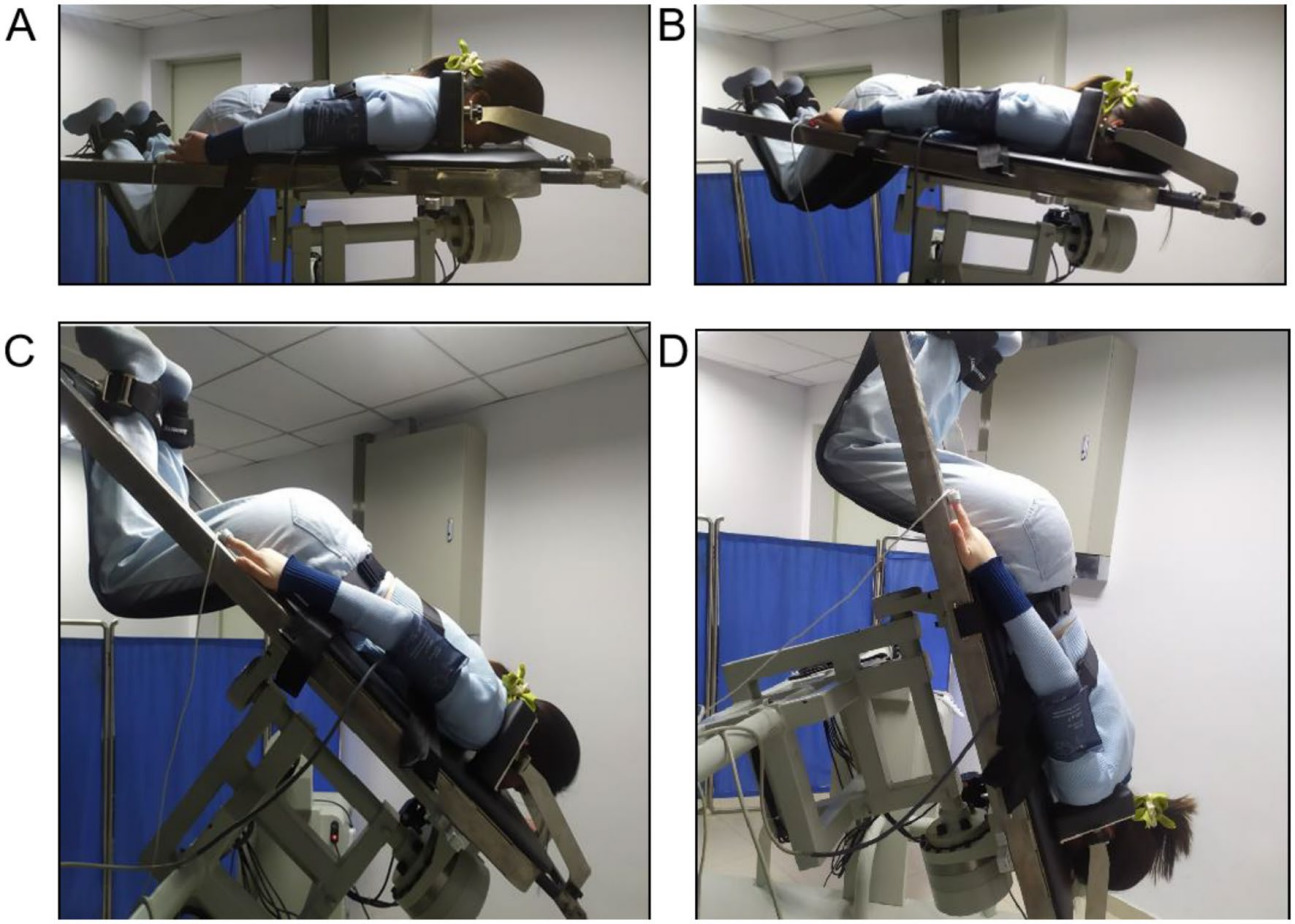
Cerebral blood flow, non-invasive intracranial pressure, and hemodynamic changes were monitored in subjects lying prone on a lithecbole bed at different angles of HDT

Subjects were positioned prone on the lithecbole bed with shoulders attached to the support frame, upper forehead attached to the bed, feet secured and straps tightened, rotated around the X axis to 0°, 10°, 40°, and 70° of HDT, and continued for 10 min (Fig. 1), and measurements were recorded in time samples lasting 90 s during the last 3 min of the steady state, with the summary values calculated by averaging the measurements, respectively. To reduce intersubject variability, all measurements were performed by a single investigator, and each subjects was required to have used the same depth and power.

### Cerebral blood flow (CBF), non-invasive intracranial pressure (nlCP), and hemodynamic measurements

Firstly, TCD-based measurements including middle cerebral artery blood flow velocity (MCAv) and/or cerebrovascular conductance coefficient (CVCi) [by dividing the MCAv by the mean arterial pressure (MAP)] are used to assess CBF at rest [22]. Measurement of cerebral blood flow velocity (MCAv) in the right and left middle cerebral arteries was performed using a TCD (Ultrasound Transcranial Doppler Flow Analyzer KJ-2V7M, Nanjing Kejin Experimental Co. Ltd., China) by placing a 2 MHz probe over the temporal window on both the right and left sides of the head at a depth of 50–60 mm.

Secondly, the right arm was fixed in a stable position and kept at the level of the heart, and blood pressure (BP), heart rate (HR), respiratory rate (RESP), and oxygen saturation (SPO_2_) were continuously monitored at the right brachial artery using a Myriad monitor (Portable Multi-Parameter Monitor PM-8000 Express, Shenzhen Myriad Biomedical Electronics Co., Ltd., China), and the electrocardiogram (ECG) was observed for any abnormalities.

The mathematical modeling approach was then used, which included the MCAv and BP-derived parameters measured by TCD as described above [23, 24], to first estimate cerebral perfusion pressure (CPPe): MAP*Vd/Vm + 14, with Vd and Vm being the diastolic phase of the MCA and the mean flow velocity, respectively, as measured by TCD. Vd and Vm are the diastolic and mean blood flow velocities of the MCA measured by TCD, respectively, and the final nlCP was calculated as MAP-CPPe.

### Data analysis

Statistical analyses were performed using SigmaPlot version 12.5 software. Data were expressed as mean ± standard deviation. Plotting was done using Grapad Prism 8.0.2 software. The Shapiro–Wilk test was used to verify the normal distribution of the data. Physiological indices at different angles (0°HDT, 10°HDT, 40°HDT, and 70°HDT) were compared using one-way repeated measures ANOVA, followed by multiple comparisons using Tukey’s test. If the variables did not obey normal distribution, a Friedman repeated-measures ANOVA was performed on the ranks, followed by Tukey’s test for multiple comparisons. *p* < 0.05 was considered significant for each statistical test.

## Results

**Fig. 2:**
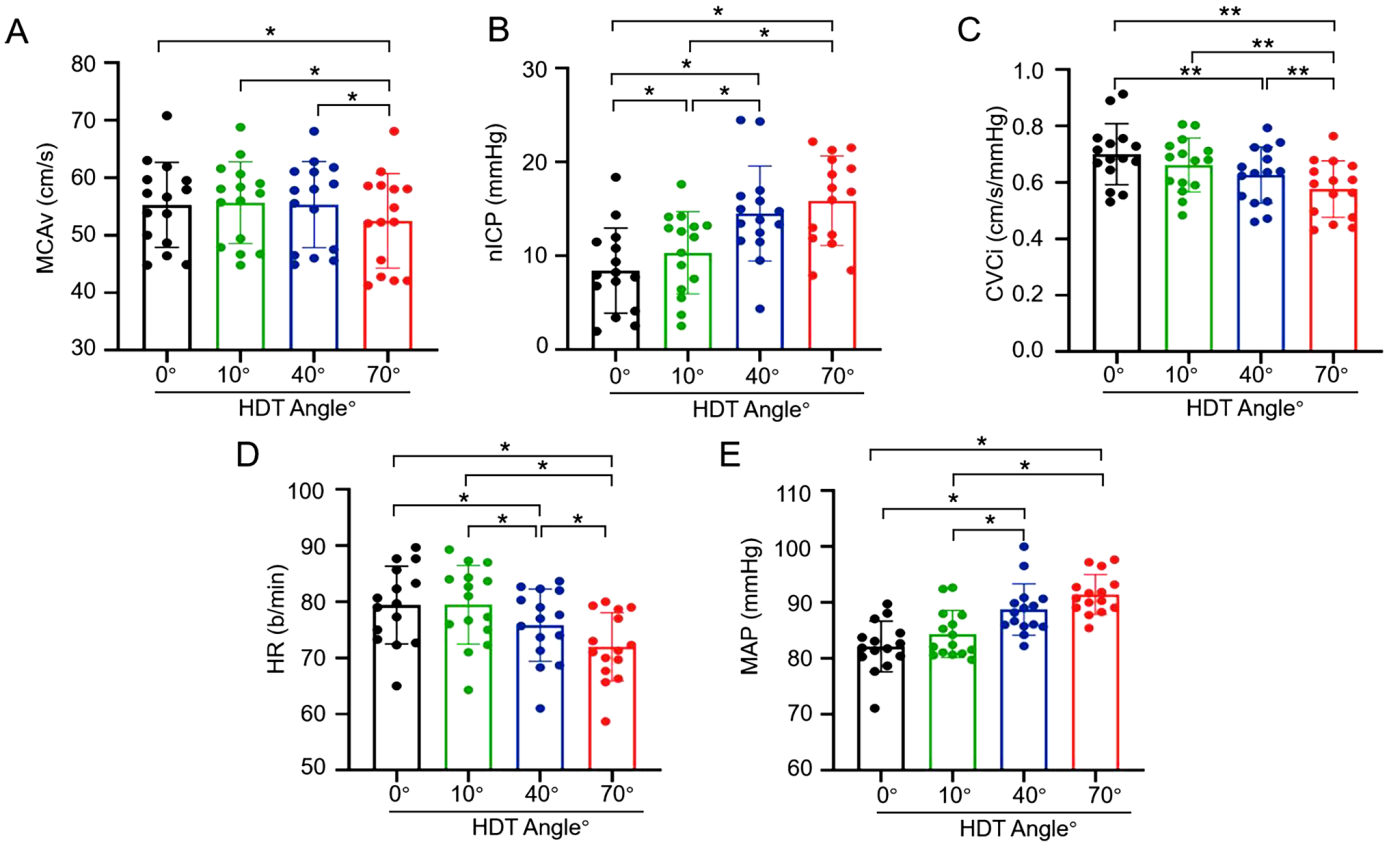
Comparison of differences between groups for each measure

The 15 volunteers included 10 males and 5 females, aged 52.07 ± 15.87 years, with a BMI of 22.9 ± 3.15 kg/m^2^, and 8 patients with previous hypertension, 5 diabetes mellitus, and 1 cerebrovascular disease (Table 1). No abnormalities were found in the RESP, SPO_2_, and ECG of the subjects throughout the experiment. Table 2 shows the results of the different measurements. Figure 2: Comparison of differences between groups for each measure.

**Table 1:** General information

Variable	Value
*Age* (*years*)	52.07 ± 15.87
*Genders*	
Male/Female	10/5
*BMI* (kg/m^2^)	22.9 ± 3.15
*History of previous illnesses* (*cases*)	
Hypertensive	8
Diabetes	5
Cardiology	0
Cerebrovascular disease	1

**Table 2:** Size of each measure of HDT at different angles

	0°HDT	10°HDT	40°HDT	70°HDT	*F*/χ^2^值	*P*值
MCAv (cm/s)	55.28 ± 7.40	55.67 ± 7.11	55.34 ± 7.48	52.53 ± 8.21^αβθ^	21.069	< 0.001^ m^
nICP (mmHg)	6.24 ± 4.49	10.33 ± 4.37^α^	14.53 ± 5.07^αβ^	15.88 ± 4.78^αβ^	27.386	< 0.001^ m^
CVCi (cm/s/mmHg)	0.68 ± 0.11	0.66 ± 0.10	0.63 ± 0.10^δ^	0.57 ± 0.10^δγΦ^	33.000	≤ 0.001^n^
HR (b/min)	79.42 ± 6.92	79.47 ± 7.00	75.82 ± 6.43^αβ^	71.98 ± 6.08^αβθ^	127.001	< 0.001^ m^
MAP (mmHg)	82.16 ± 4.54	84.38 ± 4.24	88.76 ± 4.62^αβ^	91.46 ± 3.56^αβ^	27.668	< 0.001^ m^

Values represent mean ± standard deviation. *P*-values were obtained by one-way repeated-measures ANOVA (*m*) or Friedman repeated-measures hierarchical ANOVA (*n*). α: *P* < 0.05 compared with 0°HDT (Tukey’s test). β: *P* < 0.05 compared with 10°HDT (Tukey’s test). θ: *P* < 0.05 compared to 40°HDT (Tukey’s test). δ: *P* < 0.01 compared to 0°HDT (Tukey’s test). γ: *P* < 0.01 compared to 10°HDT (Tukey’s test). Φ: *P* < 0.01 compared with 40°HDT (Tukey’s test). *MCAv* mean middle cerebral artery blood flow velocity, *nICP* noninvasive intracranial pressure, *CVCi* cerebrovascular conductance coefficient, *HR* heart rate, *MAP* mean arterial pressure, *HDT* head-down tilt

### MCAv

MCAv values changed significantly as the angle of HDT increased (F = 21.069, *P* < 0.001) and were significantly lower during 70°HDT than during 0°HDT, 10°HDT, and 40°HDT (Tukey, all *P* < 0.001).

### nICP

The nICP values increased significantly with increasing HDT angle (F = 27.386, *P* < 0.001) and were significantly higher during 70°HDT than during 0°HDT as well as 10°HDT, while nICP values were significantly higher during 40°HDT than during 0°HDT (Tukey, all *P* < 0.001). The nICP values during 40°HDT were significantly higher than 10°HDT (Tukey, *P* = 0.005). The nICP values during 10°HDT were significantly higher than 0°HDT (Tukey, *P* = 0.006).

### CVCi

CVCi values varied significantly across HDT angles (χ^2^ = 33.000, *P* < 0.001) and were significantly lower during 70°HDT than during 0°HDT, 10°HDT, and 40°HDT, while CVCi values were significantly lower during 40°HDT than during 0°HDT (Tukey, all *P* < 0.01).

### HR, MAP

Subjects’ HR values decreased significantly with increasing HDT angle (*F* = 127.001, *P* < 0.001) and were significantly lower during 70°HDT than during 0°HDT, 10°HDT, and 40°HDT, while HR values during 40°HDT were significantly lower than during 0°HDT and 10°HDT (Tukey, all *P* < 0.001). In contrast, MAP increased significantly with increasing HDT angle (*F* = 27.668, *P* < 0.001) and was significantly higher during 70°HDT than 0°HDT and 10°HDT, while MAP values during 40°HDT were significantly higher than those during 0°HDT (Tukey, all *P* < 0.001). MAP values during 40°HDT were significantly higher than those during 10°HDT (Tukey, *P* = 0.002).

## Discussions

During urological stone surgery, surgeons put patients in Trendelenburg position to facilitate surgery, but this position can lead to increased intracranial pressure and increase the risk of postoperative complications [25]. There is no relevant literature on the changes in vital signs in patients with urinary stones during postoperative active lithecbole using the inverted position, so it is important to gain an in-depth understanding of the effects on cerebral blood flow, intracranial pressure, and other vital signs during HDT at different angles.

In this study, to assess changes in cerebral blood flow, we chose the MCA for examination not only because this vessel perfuses approximately 80% of the cerebral hemispheres but also because transcranial Doppler ultrasonography is easy and repeatable to access [26]. Mean MCAv values and CVCi values were measured as an indicator of overall CBF during the inverted position. In this study, the mean MCAv values and CVCi values were statistically significantly lower between all angles of HDT, and the changes in these two metrics generally converged (Table 2). Further multiple comparisons revealed no significant differences in the mean MCAv values of the subjects during 0°, 10°, and 40° HDT (Fig. 2). These results suggest that patients with PDLS in which the inversion angle is on the gentle side can maintain relatively stable CBF during PDLS in the treatment of RFs. This is consistent with the findings of Kato et al. [27] who measured the cerebral blood flow velocity waveforms in the middle cerebral artery for the last 6 min after exposing 17 healthy subjects aged 24 ± 2 years to three angles of HDT (0° HDT, 10° HDT, and 30° HDT) for a duration of 10 min and measured the CBF using a TCD, which could be maintained at a steady state. Therefore, the authors concluded that steady-state CBF may be preserved during short-term 30° HDT [27].

In addition, Montero et al. [28], by placing 10 healthy volunteers aged 25 ± 2.1 years on 30° HDT for 3 h, found by Doppler ultrasound that cerebral perfusion was maintained during prolonged 30° HDT, regardless of changes in MAP and central venous pressure. Therefore, PDLS may have no effect on cerebral blood flow supply to patients during the treatment of RFs at a gentle tilt angle. In addition, in the present study, it was shown that the mean MCAv values were significantly reduced during 70° HDT (Fig. 2), which implies that cerebral blood flow supply was suppressed. This result is in contrast to the findings of Geinas et al. [29] who measured cerebral blood flow supply by ultrasound after exposing 21 healthy subjects aged 23.5 ± 4.6 years to 90° HDT for 10 min, and they found no significant change in the mean MCAv values during 90° HDT compared to the supine position (0° HDT). The reason for this phenomenon may be due to the wide age range of 52.07 ± 15.87 years of the 15 subjects we chose, and older patients may have problems such as vascular sclerosis, which can lead to uneven distribution of CBF [30]. These findings suggest that PDLS has a negative effect on cerebral perfusion during the treatment of RFs in an aging population [31]. Therefore, further studies are needed to elucidate whether cerebral perfusion remains unchanged during short or prolonged periods of tilting at different angles during PDLS treatment of RFs in an aging population.

In this study, we explored the changes in intracranial pressure in PDLS during the inverted position by TCD. TCD can accurately identify ICP > 20 mmHg [32], and the range of ICP monitoring can be extended by the nICP measurement technique. In addition, this noninvasive technique may offer the possibility of intracranial pressure assessment in patients with other neurological disorders. In the present study, the mean nICP values were significantly higher in the four angles of HDT and the difference was statistically significant (Table 2). This is in line with the findings of Kato et al. [27] who found that nICP values for 30° HDT were significantly higher than those for 0° or 10° HDT over a short period of time. This implies that there is a risk of increased intracranial pressure during PDLS treatment of RFs, which may be caused, on the one hand, by the fact that increased cerebral venous pressure in the sagittal sinus can directly increase ICP due to the opening of the venous system in a hydrostatic pressure gradient during HDT [33, 34]. On the other hand, the increase in ICP during HDT may also be enhanced by the increase in cerebral venous blood [35]. In this case, if the intracranial pressure continues to increase, it may lead to serious complications such as cerebral herniation, cerebral edema, respiratory failure, and coma, and the increased ICP may also lead to a decrease in cerebral blood flow, which in turn affects the normal physiological functions of the brain [36].

Multiple comparisons in this study showed statistically significant increases in mean nICP values during 0°, 10°, and 40° HDT; however, there was no significant difference in mean MCAv values during this period (Fig. 2). This result is consistent with a previous study by Piechnik et al. [37] who found that venous drainage from the head is limited during inversion and that the reduction in venous outflow may also limit the increase in arterial inflow during HDT resulting in a relatively stable CBF at rest. Based on the above studies, although the physiological elevation of ICP due to hydrostatic pressure effects and pooling of blood in the venous system is different from the mechanism of pathological elevation of ICP due to iatrogenic compression of the brain, such as head injury or hydrocephalus, the magnitude of the effects may have both direct and indirect effects on vital signs. These results suggest that the inverted position may increase the risk of increased intracranial pressure and intracranial hypertension during RFs treated with PDLS, thus requiring close monitoring of patients for changes in their condition and caution regarding the possible risk of intracranial hypertension in subjects. The results of this study may not only prevent complications of PDLS but also improve patient care for laparoscopic surgery using the Trendelenburg position [25].

In the current study, we also wanted to determine whether there were changes in HR and MAP during inversion lithecbole in PDLS. Previous studies have shown conflicting results [19–21]. Neilsen et al. [38] found that inverted body position is associated with cardiovascular risk, they investigated the effect of inverted body position on the cardiovascular function of subjects during steep HDT by exposing 26 healthy subjects with a median age of 26 years (19–56 years) to 70° and 90° HDT in a randomized order and keeping them there for 5 min, they showed that the mean MAP values were elevated during 70° and 90° HDT and no significant change in HR. Therefore, clinicians should be cautious when using inversion in patients with uncontrolled hypertension or stroke [38]. In contrast, other studies have reported no clinically significant changes in cardiovascular hemodynamics in healthy populations [19, 21]. These studies concluded that it appears to be safe for patients during inversion position [19, 21]. In the present study, MAP significantly increased and HR significantly decreased with increasing HDT angle, and the differences were all statistically significant, especially during 40° and 70° HDT (Table 2). Related literature reports that stress fluctuations in MAP may maintain a stable blood supply to the brain through autoregulatory mechanisms [39, 40]. However, if the MAP pressure is too low or too high, it may adversely affect the blood supply to the brain [41].

In addition, cardiac output is the amount of blood pumped by the heart per unit of time and is the product of HR and output per beat [42]. In the present study, the decrease in HR indicates a decrease in sympathetic nerve activity during HDT [43], which not only leads to a decrease in cardiac output but also results in a decrease in the amount of blood delivered to different parts of the body, a condition that may lead to tissue hypoxia as well as an increase in cardiac loading, which may affect brain function as well as cardiac disorders, among others. These results suggest that the use of an inverted position in PDLS during the treatment of RFs may adversely affect subjects’ cerebral perfusion. Overall, PDLS in the current study caused significant changes in blood pressure and heart rate occurrence during an inverted position, contrary to previous studies [21, 44].

There are some limitations to our study. In this study, we did not examine the effect of demographic variables (sex, age, or BMI) on cerebral blood flow, intracranial pressure, heart rate, and blood pressure in subjects treated with PDLS for RFs, even though previous similar studies have shown that the lack of certain information in the above cases may not have a direct impact on the results of the study [27–29, 38, 44, 45]. However, we are aware of the importance of collecting and analyzing demographic and comorbidity information, not only to improve the comprehensiveness and credibility of the study but also to allow for a more accurate generalization of the findings. However, we are currently ill-equipped to conduct more in-depth data analyses in subsequent experiments to consider the impact of information on these parameters on the study results, in order to gain a fuller understanding of the scope of application of the findings.

In addition, measurements using TCD only provide an indication of overall rather than local cerebral blood flow velocities and are operator-dependent. The use of MCAv obtained by TCD to assess CBF is based on previous studies and clinical experience, as it has been shown that in most cases MCA diameter is relatively stable [46], and changes in MCAv are proportional to overall CBF [47]. However, we cannot exclude the possibility that MCA diameter changes during HDT. The initial estimation of nICP was based on invasive BP waveforms in the radial or femoral arteries [48], which may have led to a less accurate estimation of nICP in this study. In addition, the cerebral blood flow autoregulation function during CO_2_ response will increase or decrease accordingly, the mechanism of which is closely related to the alteration of cerebral vascular tone. However, this study aimed to verify the safety of the PDLS procedure, and for ethical reasons, the procedure of unpercutaneous monitoring of pCO_2_ was performed in our study. This method is invasive and may cause serious complications.

## Conclusions

This study provides the necessary basis for future changes in cardiovascular function during postural lithotripsy. Our study showed that with increasing HDT angle, elevated ICP leading to increased risk of intracranial hypertension can be induced during the management of renal residual lithotripsy by PDLS, as well as a decrease in CBF leading to changes in cerebral circulation. In addition, increased BP as well as decreased HR may lead to hypoxia. The results of this study may not only prevent complications of PDLS but also improve patient care. A possible direction for future research is safer and more effective lithotripsy by lowering the HDT angle in patients with known cardiovascular disease with urinary stones.

## Data Availability

The datasets generated during and/or analyzed during the current study are available from the corresponding author on reasonable request.
